# Current Advances in Immune Checkpoint Inhibition and Clinical Genomics in Upper Tract Urothelial Carcinoma: State of the Art

**DOI:** 10.3390/curroncol29020060

**Published:** 2022-01-29

**Authors:** Gianluigi Califano, Idir Ouzaid, Paul Laine-Caroff, Arthur Peyrottes, Claudia Collà Ruvolo, Benjamin Pradère, Vincent Elalouf, Vincent Misrai, Jean-François Hermieu, Shahrokh F. Shariat, Evanguelos Xylinas

**Affiliations:** 1 Urology Unit, Department of Neurosciences, Reproductive Sciences and Odontostomatology, Federico II University of Naples, 80131 Naples, Italy; gianl.califano2@gmail.com (G.C.); c.collaruvolo@gmail.com (C.C.R.); 2 Department of Urology, Bichat-Claude Bernard Hospital, Assistance-Publique Hôpitaux de Paris, Paris University, 75018 Paris, France; idir.ouzaid@aphp.fr (I.O.); paul_laine@live.fr (P.L.-C.); peyrottesarthur@gmail.com (A.P.); jean-francois.hermieu@aphp.fr (J.-F.H.); 3 Department of Urology, Medical University of Vienna, 1090 Vienna, Austria; benjaminpradere@gmail.com (B.P.); shahrokh.shariat@meduniwien.ac.at (S.F.S.); 4 Department of Urology, Hospital Claude Galien, 91480 Quincy-sous-Sénart, France; vincentelalouf@gmail.com; 5 Department of Urology, Clinique Pasteur, UMR-1048 Toulouse, France; v.misrai@clinique-pasteur.com; 6 University of Paris, INSERM, Immunologie Humaine Physiopathologie & Immunothérapie, F-75010 Paris, France

**Keywords:** upper tract urothelial carcinoma, prognosis, immune check point inhibition, radical nephroureterectomy, genetics

## Abstract

Upper tract urothelial carcinoma (UTUC) is a rare and challenging-to-treat malignancy. In most patients it is a sporadic tumor entity, less commonly it falls on the spectrum of Lynch syndrome, an autosomal dominant familial tumor syndrome. Localized UTUC with high-risk features as well as the metastatic disease scenario might require systemic therapy. Platinum-based combination chemotherapy is currently the recommended management option. However, the introduction of immune checkpoint inhibitors into the therapeutic armamentarium has led to a paradigm shift in treatment standards. Immunotherapy has been shown to be safe and effective in treating at least metastatic UTUC, although UTUC-specific high-level evidence is still lacking. Recent technological advances and noteworthy research efforts have greatly improved the general understanding of the biological landscape of UTUC. According to the main findings, UTUC represent a particular subtype of urothelial carcinoma frequently associated with activated FGFR3 signaling, a luminal–papillary phenotype and a T-cell-depleted microenvironment. This improved knowledge promises precision oncology approaches that match treatment decision strategies and genomic profile to ultimately result in better clinical outcomes. The aim of this review was to summarize the main currently available evidence on immune checkpoint inhibition and clinical genomics in UTUC.

## 1. Introduction

Upper tract urothelial carcinoma (UTUC) is a rare and challenging-to-treat malignancy. It defines urothelial carcinomas (UCs) with primary pyelocaliceal and ureteral tumor location. Although UTUC differs in terms of biological and clinical features from urothelial carcinoma of the bladder (UCB), it is often addressed based on data extrapolated from UCB studies, due to the limited high-level UTUC-specific evidence [1].

UTUC exhibits aggressive clinical behavior. At the time of diagnosis, more than half of patients with UTUC harbor muscle-invasive disease, and up to one in ten are already metastatic [2]. UTUC is also associated with poor clinical outcomes, with 5-year specific survival less than 50 and 10% for pT2/T3 and pT4 tumor stages, respectively [1]. Therefore, non-metastatic UTUC with high-risk features is already considered an early systemic disease [3]. Radical nephroureterectomy (RNU) still represents the mainstay of treatment for patients with high-risk localized UTUC. Based on the currently available literature, the addition of platinum-based combination chemotherapy, to definitive surgery has been shown to improve the prognosis compared to RNU alone [4,5,6] and constitutes the current gold standard treatment according to international guidelines.

Metastatic UTUC is a deadly disease. Platinum-based combination chemotherapy remains the recommended management strategy in the first-line setting of the metastatic scenario. However, the introduction of immune checkpoint (IC) inhibition has determined a paradigm shift in treatment standards [7].

Major advances in the understanding of UTUC biology have been provided by recent genomic and gene expression studies [8]. This improved knowledge promises precision oncology approaches that match treatment decision strategies and genomic profile to ultimately result in better clinical outcomes. Additionally, light has been shed on novel pathways for therapeutic targeting [9].

This review summarizes the main currently available evidence on IC inhibition and clinical genomics in UTUC.

## 2. Immune Checkpoint Inhibition

Immunotherapy is changing the way we think about and treat UC [7,10,11]. Addressing a high decades-old unmet medical need, the PD-1/PD-L1 pathway inhibition has been shown to be safe and effective in the treatment of metastatic UC. Furthermore, the inhibition of IC CTLA-4 in different stages of the disease is being investigated [11,12]. However, immunotherapy for UTUC still lacks specific evidence. The available data are in fact extrapolated from UC studies, regardless of the primary tumor location in the upper or lower urothelium.

The use of immunotherapy, through IC inhibitors (ICIs), for the therapeutic management of UTUC is very intriguing. Indeed, UTUC remains a challenging to treat tumor due to several aspects [6]. First, patients with UTUC are commonly elderly and frail. Second, both high-risk localized and metastatic diseases might require systemic therapy. Third, most patients have decreased renal function at baseline (and even more after surgery due to the loss of a renal unit) and are unfit for nephrotoxic therapies. Fourth, the difficulties linked to the preoperative tumor assessment in localized disease increase the risk of overtreatment in patients with non-muscle invasive disease [6,7]. Taken together, these observations highlight the need for a therapeutic strategy in UTUC patients that should be effective and associated with a low toxicity profile.

Table 1 summarizes the main currently available data on immunotherapy in UTUC.

### 2.1. High-Risk Localized UTUC

RNU remains the cornerstone of treatment for patients with localized UTUC and high-risk features. Recent evidence points to the significant survival benefit of adding systemic therapy, in the form of platinum-based chemotherapy, compared to surgery alone. Currently, the evidence for adjuvant chemotherapy appears stronger (with positive level 1 evidence) than that for neoadjuvant chemotherapy (at best level 2 evidence) [21,22]. Accordingly, the 2021 European Association of Urology (EAU) guidelines strongly recommend the use of post-operative systemic platinum-based chemotherapy in patients with muscle-invasive UTUC [1].

The treatment scenario of non-metastatic muscle-invasive bladder carcinoma (MIBC) has been revolutionized by recent studies supporting the use of ICIs. Short courses of single-agent neoadjuvant pembrolizumab prior to radical cystectomy were shown to be safe and effective, with 42% ypT0 and 54% yp < T2 responses for MIBC patients, in the PURE-01 trial [23].

Following this exciting success, the PURE-02 study, a feasibility study of neoadjuvant pembrolizumab in patients with UTUC, was carried out [13]. Ten patients with non-metastatic UTUC presenting high-risk features according to the modified EAU definition were enrolled [1]. Overall, five, three, and two patients harbored UTUC in the ureters, renal pelvis, and both sites for unilateral multifocal involvement, respectively; seven patients (70%) were male. Treatment consisted of three courses of 200 mg intravenous pembrolizumab every 3 weeks, followed by RNU within 14 days of the last dose. One (10%) treatment-related death occurred within a month of the first pembrolizumab course due to the development of pneumonia and septic shock. Nine patients (90%) completed the neoadjuvant course. One (14.3%) patient achieved a clinical and radiological complete response and refused to undergo RNU. However, her follow-up is still too immature to draw appropriate conclusions. The remaining cases were characterized as uncertain responses or overt nonresponses to the neoadjuvant treatment. Indeed, two (20%) had disease progression at clinical restaging and received subsequent cisplatin-based chemotherapy, prior to RNU.

Although the sample size was very small, the authors of the PURE-02 study did not observe promising signals of activity from single-agent pembrolizumab in the neoadjuvant treatment of UTUC. Possible reasons for the conflicting results reported by the PURE-01 and PURE-02 studies on the preoperative efficacy of pembrolizumab in lower and upper urinary tract UC, respectively, may lie in the intrinsic biological diversity of tumors based on primary location along the urothelium. Indeed, UTUC exhibits a unique mutational and gene expression profile, which will be carefully analyzed below.

Finally, the efficacy and tolerability of immunotherapy, specifically durvalumab agent, in combination with chemotherapy in the neoadjuvant setting of high-risk localized UTUC patients is currently being investigated in phase II [24] and phase III [25] clinical trials. However, these mentioned studies are still in recruiting status, and no results have been published yet.

### 2.2. First-Line Metastatic UTUC

Cisplatin-based chemotherapy has been the first-line treatment for patients with metastatic UC for over three decades, regardless of the primary tumor location in the lower or upper urinary tract. This systemic disease management strategy conferred an overall survival (OS) benefit of 9–15 months, but at the cost of a significant burden of toxicity [26]. However, in real life, up to two-thirds of UC patients result in being unfit for cisplatin, due to impaired performance status or comorbidities, and alternative chemotherapy approaches are associated with a short duration of response, poor survival, and high toxicity [27,28].

The use of the ICIs atezolizumab and pembrolizumab has been approved for the treatment of PD-L1-positive patients with metastatic UC ineligible for cisplatin-based first-line chemotherapy, based on the results of the phase II trials IMvigor 210 and KEYNOTE-052 [14,15].

Atezolizumab was associated with a median OS benefit of 15.9 months in the cisplatin-unfit patient cohort with metastatic UC included in the IMvigor 210 study (median follow-up 17.2 months) [14]. The proportion of patients with UTUC (*n* = 33, 28%) who achieved a complete or partial response, defined as objective response rate (ORR), was 39%. Furthermore, the treatment toxicity profile has proved to be largely acceptable even considering that more than 70% of the study population showed an impairment of the renal function at baseline.

Pembrolizumab was associated with an ORR of 22% in 69 (19%) cisplatin-unfit patients with metastatic UTUC included in the KEYNOTE-052 study [15]. In the overall cohort, a PD-L1 positivity, defined as a combined positive score (CPS) ≥ 10% was related to a greater treatment response rate, with an ORR of 38%. Again, Pembrolizumab showed an acceptable toxicity profile in this disease setting.

The addition of atezolizumab and pembrolizumab to platinum-based chemotherapy in the management of metastatic UC has been investigated in two phase III randomized controlled trials (RCT), the IMvigor 130 and KEYNOTE-361, including 312 (26%) and 64 (18%) patients with UTUC, respectively [16,17]. The combination of immune checkpoint inhibition with standard-of-care chemotherapy did not show significant improvement in OS compared to chemotherapy alone, as a first-line strategy in the metastatic setting of UC.

### 2.3. Second-Line Metastatic UTUC

Long-term remissions after platinum-based chemotherapy for patients with metastatic UC are reported in up to 10% of patients [26]. Consequently, disease progression on platinum is a frequent occurrence. The second-line management of platinum-pretreated patients with metastatic UTUC remains challenging. Furthermore, patients have already borne a significant burden of treatment-related toxicity.

The use of the ICIs pembrolizumab, atezolizumab, avelumab, nivolumab and durvalumab has been approved for the treatment of patients with metastatic UC who have progressed during or after previous platinum-based combination chemotherapy [7]. However, contrary to pembrolizumab, recently, the Food and Drug Administration no longer approves the use of atezolizumab in this setting [29]. Moreover, durvalumab and nivolumab were tested in phase I and II studies that did not report any subgroup analyses based on primary tumor location [30,31,32].

According to the results of the phase III RCT KEYNOTE-045 trial, pembrolizumab was the first ICI supported by level 1 evidence for the treatment of UC platinum-refractory patients [18]. Over a median follow-up of 14.1 months, pembrolizumab was associated with a significant OS benefit compared to investigator/institution’s choice chemotherapy in the second-line UC management setting. In those patients with UTUC (*n* = 75, 13.8%), the pembrolizumab treatment was associated with a reduction in the risk of death of 50%. Additionally, the ORR was 21.1% with pembrolizumab compared with 11.4% in the chemotherapy group (*p* = 0.001). These findings were further confirmed after two years of follow-up [33].

Atezolizumab was associated with an ORR of 8% in 65 (21%) patients with metastatic UTUC included in the platinum-pretreated cohort of the phase II IMvigor 210 trial [19]. Interestingly, the atezolizumab treatment showed durable activity related to the PD-L1 expression on immune cells in patients with metastatic UC, with a 26% of ORR in the group of those overexpressing PD-L1 vs. 15% in the overall population. This study was even the first pointing out the association of TCGA subtypes with the response to ICIs in UC, highlighting the tumor mutation load as an efficacy biomarker.

Avelumab was associated with an ORR of 11% in 36 (22%) patients with metastatic platinum-refractory UTUC and at least 6 months of follow-up included in the phase I JAVELIN study [20].

## 3. Clinical Genomics in UTUC

Precision oncology aims to functionalize genomics in the therapeutic management of cancer patients. Significant efforts have been and continue to be made to understand the mechanisms that drive carcinogenesis in order to implement the personalized treatment pathway selection process and lead to better patient outcomes. Furthermore, it has allowed for the ability to identify biomarkers for clinical response [9].

UCs respond differently to standard-of-care treatment strategies depending on the location of the primary tumor [34]. This simple observation underlies potential differences in mutational and expression profiles of UCs with significant clinical implications. In fact, UCB and UTUC share purely histological appearance, differing instead in embryological, biological and practical aspects [35]. More interestingly, renal pelvis and ureteral UCs, conventionally joined as UTUC, also differ in genomics and clinical management [8].

Recent technological advances have led to a better understanding of the mutational and gene expression profiles of UCs. Although several studies have depicted the genomic landscape of UCB, knowledge of UTUC remains more limited. Again, UTUC pay the price for a lower epidemiological frequency compared to their bladder counterpart.

**Figure 1:**
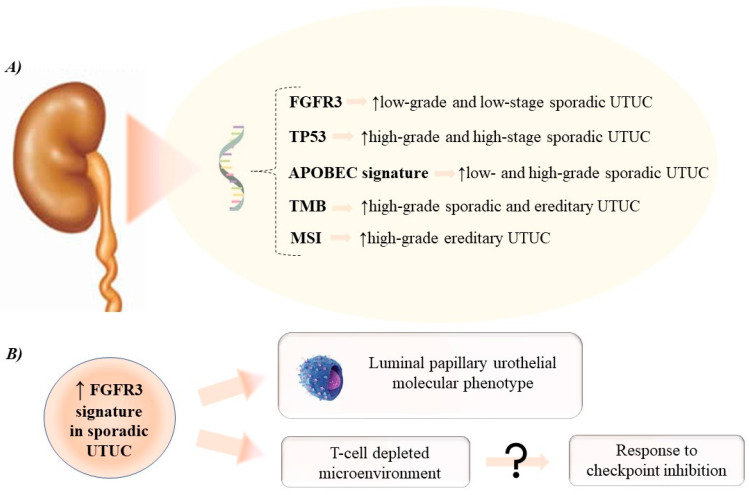
Overview of mutational (**A**) and gene expression (**B**) profile in upper tract urothelial carcinoma. APOBEC: apolilpoprotein B mRNA editing enzyme catalytic polypeptide-like; FGFR3: fibroblast growth factor receptor 3; MSI: microsatellite instability; TMB: tumor mutational burden; TP53: tumor protein 53; UTUC: upper tract urothelial carcinoma.

Figure 1 provides an overview of mutational and gene expression profile of UTUC.

### 3.1. Mutational Profile of UTUC

Sporadic UTUC shares mutational features in similar genes and epigenetic genes with UCB, but at varying frequencies. Furthermore, genetic patterns differ according to the tumor stage and grade, both for UTUC and UCB. Lastly, different genetic profiles have been reported between UCs arising in renal pelvis vs. ureters.

Using next-generation sequencing, FGFR3 is recognized as the most frequently mutated gene in UTUC, with mutational rates ranging from 40% to 80% in sporadic tumors [36,37,38]. Most FGFR3 mutations are missense. Other common alterations are described for oncogenes or tumor suppressor genes, such as HRAS and genes involved in the p53 signaling (TP53, ATM, ATR) [39]. TP53 mutations are reported in up to 30% of UTUC. Moreover, UCs, regardless of their location in the urothelium, share a molecular signature attributed to the apolilpoprotein B mRNA editing enzyme catalytic polypeptide-like (APOBEC) family of cytosine deaminases, which converts cytosine to uracil [40]. The result is single-stranded DNA editing. Evidence showed that a high APOBEC signature mutation burden was strongly correlated with the tumor mutational burden (TMB) [36].

FGFR3 mutations are more common in low-grade and low-stage UCs. In UTUC, the incidence of FGFR3 mutations is up to 80% in low-grade tumors, while varying between 15 and 30% in their high-grade counterparts [34]. These observations are also common for UCB, where alterations in FGFR3 are more frequently reported for non-muscle-invasive bladder carcinoma (NMIBC) compared to muscle-invasive bladder carcinoma (MIBC) and particularly for low-grade NMIBC [34]. In addition, FGFR3 mutations are correlated with a better clinical prognosis. Conversely, patients with high-grade and high-stage UC appear more likely to harbor TP53 mutations. Indeed, TP53 gene alterations are the most commonly reported in MIBC, and generally correlate with more aggressive UCs and worse clinical outcomes [41].

Necchi et al. used comprehensive genomic profiling to show that FGFR3 and HRAS gene alterations were more common in UTUC compared to UCB [8]. More interestingly, the mutational frequency differences of these two genes have been reported based on the location of the primary tumor in the renal pelvis relative to the ureters. The authors reported alterations in FGFR3 and HRAS in 28 vs. 22% and 9.5 vs. 1.8% in the renal pelvis compared to ureteral tumors, respectively.

UTUC is also represented in the spectrum of Lynch syndrome, an autosomal-dominant familiar cancer syndrome [1]. Lynch-related UTUC harbor germline mutations in the DNA mismatch repair (MMR) genes, with the majority of tumors developing in MSH2 mutation carriers. Loss of function in the MMR system results in microsatellite instability (MSI) throughout the genome. MSI have been found in up to 20% of UTUC, as compared to less than 1% in UCB [42]. In addition, high MSI correlates with a higher TMB and better clinical prognosis.

### 3.2. Gene Expression Profile of UTUC

Several studies have addressed the comprehensive gene expression profile of UCs, mainly focusing on UCB. Five molecular subtypes of MIBC were identified in The Cancer Genome Atlas (TCGA) classification, through a combination of expression and clinical data [43]. Luminal–papillary, basal–squamous, luminal–infiltrated, luminal, and neuronal were the subtypes reported in 35, 35, 19, 6, and 5% of MIBC, according to TCGA, respectively. From a clinical-prognostic point of view, the luminal–papillary and neuronal subtypes have the best and worst survival outcomes, respectively. To overcome the classification schemes proposed later on and provide a consensus definition, Kamoun et al. recently described six biological classes of MIBC: luminal papillary (LumP), luminal nonspecified, luminal unstable (LumU), stroma-rich, basal/squamous (Ba/Sq), and neuroendocrine-like (NE-like) [44]. All luminal subtypes overexpress urothelial differentiation markers, LumP tumors have an FGFR3 signature, while Ba/Sq tumors overexpress basal markers.

Again, data on UTUC remains scarce, mainly due to its rarity. UCB and UTUC appear to share similar gene expression profiles, but at varying frequencies [34]. In this context, UCB tends to express genes that mark urothelial basal cells and belongs to the basal-like subtype, while most UTUC expresses genes consistent with a luminal urothelial molecular subtype. Even more interesting is the observation that an UCB developing after UTUC is likely to be luminal, while an UTUC arising after UCB is often basal.

The luminal expression profile of UTUC was confirmed and refined by Robinson et al. [38]. Through RNA sequencing of 32 UTUC tumors, the authors found that 84.3% (*n* = 24) of their samples were luminal tumors and 62.5% (*n* = 20) showed a luminal–papillary phenotype, using the TCGA subtypes. In addition, examining the expression of key immune genes, 87.5% (*n* = 28) of UTUC tumors were found to be T-cell depleted with downregulation of the T-cell-related signaling. The authors concluded that UTUC represents a particular subtype of UC associated with a luminal–papillary phenotype and T-cell-depleted microenvironment [38].

### 3.3. Implications for Systemic Therapy

The efficacy of systemic cancer therapy ultimately relies on the tumor genomic landscape. Advances in the knowledge of the biological profile of UTUC promise precision therapeutic management. Above all, efforts to investigate the mutational and gene expression aspects of UTUC have been noteworthy, even considering that this is often managed on the basis of UCB evidence, due to its rarity.

Platinum-based chemotherapy is currently the systemic approach of choice in both high-risk localized and metastatic UTUC [1]. It works by inducing DNA damage and promoting cellular apoptosis. A high TMB, supported by a mutation profile in TP-53 signaling or a high MSI, defines the biological identikit of UTUC that should be associated with a higher response rate to chemotherapy [45].

Immunotherapy, through the PD-1/PD-L1 pathway inhibition, has been shown to be safe and effective in treating at least metastatic UTUC [7]. It works by promoting the defense of the immune system. Again, a high TMB is associated with the expression of surface neoantigens on tumor cells and a high response rate to immunotherapy [46].

Most sporadic UTUC show mutations in the FGFR3 gene. Often, these are associated with a better prognosis. Therapeutic strategies selectively targeting the FGF receptor could be addressed to FGFR3 mutated tumors. Erdafitinib is a pan-FGF tyrosine kinase inhibitor approved as second-line treatment for locally advanced or metastatic UC with FGFR mutations [12]. In a recent phase II study, the use of erdafitinib was associated with a 40% response rate in 99 patients with disease progression following chemotherapy [47]. This study included 23 patients with UTUC and visceral metastases showing a 43% response rate. Additional FGFR-directed agents also continue to be investigated across multiple disease stage in mutated UC including infigratinib and rogaratinib among others [12]. Furthermore, ongoing trials are combining these agents with ICI and chemotherapy regimens.

Overall, UTUC exhibits a landscape of more than 50% possibly actionable genomic alterations [8]. A field of growing interest among investigators is the use of prognostic biomarkers to identify targetable biological profiles. Blood-based evaluation, using ct-DNA, of genomic signatures may present a further opportunity to develop response biomarkers for available therapeutic armamentarium options. In UCs, proof-of-concept data documented that ctDNA is detectable in plasma and urine, and could be a prognostic factor [48,49,50,51]. Liquid biopsy could represent a cost-effective and minimally invasive method for biomarker identification and patient stratification.

## 4. The Immune Microenvironment in UTUC

The immune defense system plays a leading role in the host’s antitumor response. This occurs mainly through the recognition of neoantigens exposed on the tumor cell surface, as a consequence of the genomic alterations that promote carcinogenesis. In particular, CD8 T cells support the immuno-elimination of tumor cells [52].

Tumors differ in immunogenicity levels, and therefore in the tumor immune microenvironment. Consequently, different tumors exhibit different immunological potential to counteract the development and progression of the disease. Overall, UCs have long been recognized as being strongly immunogenic tumors [10].

Immuno-editing is defined as the path by which the tumor bypasses immune-surveillance. Activation of T-cell function inhibitory pathways, such as the PD-1/PD-L1 IC axis, is a well-known tumor immuno-evasion strategy. Indeed, this pathway has been shown to play an important role in the development of UC [53].

Taken together, these observations provide the rationale for the clinical investigation of cancer immunotherapy, through IC inhibition, in UC at the beginning of the last decade. At that time, the use of antibodies inhibiting the PD-1/PD-L1 pathway had already demonstrated robust anti-cancer activity in several malignancies [54].

Overall, immunotherapy with ICI has been shown to be effective in treating UC [7].

UTUC display different mutational and gene expression profiles compared to UCBs. It follows that the tumor immune microenvironment may also differ among UC based on the primary tumor location. Actually, sporadic UTUC has a luminal–papillary T-cell-depleted contexture and activated FGFR3 signaling. In addition, upregulation of FGFR3 in UTUC seems to be associated with a lower CD8 T-cell gene signature and, more interestingly, it has been shown to be important in shaping the observed T-cell-depleted phenotype [38]. Consequently, sporadic UTUC should frequently be characterized by an immune desert profile and refractoriness to immunotherapy. In contrast, UTUC developed in a Lynch syndrome context exhibits high MSI and TMB. According to these biological features, it could be considered an immune hot tumor. However, the profiles depicted did not match the clinical outcomes reported in the leading studies investigating the efficacy of immunotherapy in UTUC [7]. Again, this is indirect evidence that a significant knowledge gap has yet to be filled.

## 5. Conclusions

UTUC is a challenging-to treat malignancy, also due to its rarity and limited high-level evidence to support its clinical management. However, significant advances in knowledge have recently been made to ultimately improve patient prognosis. Immunotherapy is changing the way we think about and treat UC, and PD-1/PD-L1 pathway inhibition has been shown to be safe and effective in the systemic management of metastatic UTUC. Currently, therapeutic decision making remains primarily guided by clinical and radiological evaluation. Improved understanding of the genomic landscape of UTUC should lead to a paradigm shift in treatment strategies. Several therapeutically actionable molecular pathways have recently been described, and novel clinical trials investigating therapeutic options for UTUC by stratifying patients for the biological profile are expected.

## Figures and Tables

**Table 1:** Main currently available data on immune checkpoint inhibition in UTUC.

Drug	IV Dosage and Timing	Target	Trial	Study Design	UTUC Population, *n* (%) *	Primary Tumor Location, *n* (%)	ORR % ^ǂ^
***High-risk localized UTUC—neoadjuvant setting***							
**Pembrolizumab**	200 mg/3 wks × 3	PD-1	PURE-02 [13]	Single arm	10 (100)	5 (50) renal pelvis 3 (30) ureter 2 (20) bilateral	14.3 ^†^
***Metastatic UTUC—first line setting***							
**Atezolizumab**	1200 mg/3 wks	PD-L1	IMvigor 210 [14] Cohort 1	Phase II Single arm	33 (28)	n/a	39
**Pembrolizumab**	200 mg/3 wks	PD-1	KEYNOTE-052 [15]	Phase II Single arm	69 (19)	n/a	22
**Atezolizumab**	1200 mg/cy	PD-L1	IMvigor 130 [16]	Phase III Triple arm RCT	312 (26)	175 (56) renal pelvis 137 (44) ureter	n/a
**Pembrolizumab**	200 mg/3 wks	PD-1	KEYNOTE-361 [17]	Phase III Triple arm RCT	231 (23)	n/a	n/a
***Metastatic UTUC—second line setting***							
**Pembrolizumab**	200 mg/3 wks	PD-1	KEYNOTE-045 [18]	Phase III Two-arm RCT	75 (14)	n/a	n/a
**Atezolizumab**	1200 mg/3 wks	PD-L1	IMvigor 210 [19] Cohort 2	Phase II Single arm	65 (21)	42 (65) renal pelvis 23 (35) ureter	8
**Avelumab**	10 mg/kg/2 wks	PD-L1	JAVELIN [20]	Phase I Single arm	36 (22)	n/a	11 ^#^

IV: intravenous; n/a: not available; ORR: objective response rate; PD-1: Programmed Death-1; PD-L1: Programmed Death-Ligand 1; RCT: randomized controlled trial; UTUC: upper tract urothelial carcinoma. * The percentage is relative to the overall urothelial carcinoma patients enrolled in the study trial. **ǂ** The percentage is only referred to patients with UTUC. **^†^** Defined as radiological and endoscopic complete response. ^#^ UTUC patients with at least 6 months of follow-up.
